# Investigation of different electrochemical cleaning methods on contaminated healing abutments in vitro: an approach for metal surface decontamination

**DOI:** 10.1186/s40729-020-00265-z

**Published:** 2020-11-08

**Authors:** Thiha Tin Kyaw, Takao Hanawa, Shohei Kasugai

**Affiliations:** 1 Department of Oral Implantology and Regenerative Dental Medicine, Division of Oral Health Sciences, Graduate School of Medical and Dental Sciences, Tokyo Medical and Dental University, 1-5-45 Yushima, Bunkyo-ku, Tokyo, 113-8510 Japan; 2 Department of Metallic Biomaterials, Institute of Biomaterials and Bioengineering, Tokyo Medical and Dental University, Tokyo, Japan

**Keywords:** Decontamination, Detergent, Electrolysis, Healing abutments, Sodium bicarbonate

## Abstract

**Background:**

To evaluate the effects of electrolysis on cleaning the contaminated healing abutment surface and to detect the optimal condition for cleaning the contaminated healing abutment.

**Methods:**

Ninety healing abutments removed from patients were placed in 1% sodium dodecyl sulfate solution and randomly divided for electrolysis with 7.5% sodium bicarbonate in the following three different apparatuses (*N* = 30): two stainless steel electrodes (group I), a copper electrode and a carbon electrode (group II), and two carbon electrodes (group III). The samples were placed on cathode or anode with different electric current (0.5, 1, and 1.5 A) under constant 10 V for 5 min. Electrolyte pH before and after electrolysis were measured. Then, the samples were stained with phloxine B and photographed. The proportion of stained areas was calculated. The surface was examined with a scanning electron microscope (SEM) and energy-dispersive X-ray spectroscopy (EDS).

**Results:**

Electrolyte pH decreased after electrolysis at 1 A and 1.5 A in group I and II. Applying cathode at 1 A in group III, the amount of residual contamination was the lowest in all the conditions examined in the present study. SEM images revealed that applying cathode at 1.5 A in group I induced a rough surface from the smooth surface before the treatment. EDS analysis confirmed that the surfaces treated on cathode at 1 A in group III revealed no signs of organic contamination.

**Conclusion:**

Electrolysis of using carbon as electrodes, placing the contaminated healing abutments on cathode, and applying the electric current of 1 A at constant 10 V in 7.5% sodium bicarbonate could completely remove organic contaminants from the surfaces. This optimized electrochemical cleaning method seems to be well worth investigation for the clinical management of peri-implant infections.

## Background

A healing abutment is a small metal cap placed on the dental implant. In dental implant treatment, a healing abutment is first placed on the implant. The top of the healing abutment is exposed in the oral cavity, while its body penetrates the soft mucosal tissue. During the implant treatment, the healing abutment is temporarily removed and replaced into several times until the prosthesis is delivered. During these procedures, the clinician can always observe the contaminated surface of the healing abutment.

Although the clinician cleaned the contaminated healing abutment with conventional cleaning methods such as mechanical wiping with disinfecting clothes or ultrasonic bath for 10–60 min in alcohol or water before replacing it again, the surface remains dirty after cleaning. Thus, new cleaning method using a detergent and a strong solvent was developed to clean the contaminated surface [1]. Although this new method can effectively clean the surface, the perfect cleaning was not achieved. In addition, the procedure of this cleaning method is neither simple nor safe.

Peri-implantitis, an inflammatory destruction of the tissues around the implant, is a big clinical problem in dentistry. Colonization of bacterial biofilm on dental implant surface is scientifically accepted as the main reason for peri-implantitis [2, 3]. Hence, most of the treatments for peri-implantitis are based on the treatment of periodontitis [4]. However, the form and surface structure of the dental implant and the tooth differ greatly. Current implant surface is not smooth because of being modified to enhance osseointegration. Biofilm tightly adheres to the implant’s rough surface. Furthermore, the implant has threads and grooves. These features make the mechanical cleaning imperfect [5].

Most of the current decontamination methods have been focused on the elimination of the bacteria without any physiochemical alteration of the implant surface or removal of the organic contaminants that tightly adhered to titanium surfaces [6]. Interestingly, an innovative approach to disinfect the implants by electrolysis has been recently reported [7]. This alternative minimally invasive approach effectively reduced the number of viable microorganisms on the dental implants. Pure water can be decomposed into hydrogen and oxygen. At the cathode, water is decomposed into hydrogen and hydroxide ions, which creates an alkaline environment of high pH. At the anode, oxygen and protons are generated producing low pH condition. In addition, oxidative substances are formed depending on the material of the electrode [8] and current/voltage.

Recently, disinfection of biofilm-contaminated implant surfaces with low direct currents has been reported [9, 10]. In these previous studies, electrolysis was applied to kill or remove the bacteria forming the oral biofilm. Charging the implant afflicted by bacteria with current or voltage is extremely effective in particular concerning the removal of organic residues still adhering on the material after the bacteria have been killed. The purpose of the present study was to examine the effects of electrolysis on cleaning the contaminated healing abutment surface and to detect the optimal condition for cleaning the contaminated surface.

## Materials and methods

Ninety healing abutments removed from patients at the Dental Implant Clinic, Dental Hospital, Tokyo Medical and Dental University, were used. As this clinical study is an in vitro experimental study, the university ethical committee decided that ethical approval was not necessary.

These healing abutments were at least for 4 weeks up to 6 weeks in patients’ oral cavities. All the healing abutments were for the implants of Nobel Biocare. The following two solutions were prepared: 1% sodium dodecyl sulfate solution (SDS solution, Fujifilm Wako Pure Chemical Corp., Tokyo, Japan) and 7.5% sodium bicarbonate solution (NaHCO_3_, LEC Chemical Corp., Tokyo, Japan).

### Electrochemical cleaning

Three different electrochemical apparatuses with the two-electrode electrochemical cell was set up as follows: group I, two stainless steel (TS 200, Iwata Manufacturing Co. Ltd., Seki, Gifu) electrodes; group II, a copper electrode (Pure Copper Type 26784, Shimomura Corp., Kowloon, Hong Kong) as a working electrode and a carbon electrode (Carbon Rod, Sano Factory, Tokyo, Japan) as a counter electrode; and group III, a carbon electrode (Carbon plate, Sano Factory, Tokyo, Japan) as a working electrode and a carbon electrode (Carbon Rod, Sano Factory, Tokyo, Japan) as counter electrode. All the electrodes were immersed in an electrolyte solution and the samples were placed on the flat electrode placed at the bottom of the electrolytic chamber with a customized connector at room temperature. Electrochemical tests were conducted in all cell types. The samples served as anode or the cathode in all chambers used in this study for electrolysis. The glass chamber was mounted and 600 ml of electrolyte of 7.5% NaHCO_3_ was poured into the chamber. The fresh electrolyte was used at each electrolysis. In group I, one stainless electrode was a working electrode, where the samples were placed, and another stainless-steel electrode as a counter electrode. In group II, the copper electrode was used as a working electrode and carbon electrode was used as a counter electrode. In group III, carbon electrodes were used as a working electrode and a counter electrode. Voltage was applied with an electric power supply (ATTO Crosspower 500, ATTO Corp., Tokyo, Japan). Applied voltage was constant at 10 V and three different direct currents of 0.5 A, 1 A, and 1.5 A were applied for 5 min to induce the electrochemical (oxidation/reduction) reactions. These reactions can generate the oxidative species that remove and inactivate bacteria.

The major reaction at the cathode is the reduction of water or the reduction of oxygen.

Oxidation 2H_2_O + 2e- = H_2_ + OH-

Reduction O_2_ + 4e- + 2H_2_O = 4OH-

Immediately after removal from the patient’s mouth, the contaminated healing abutments were placed in 1% SDS solution into 20 min and randomly divided for cleaning with the three different electrolytic groups: group I, group II, and group III. Keeping a confidence level “alpha” 0.05 and power of the study 90%, the sample size was estimated to be 30 in each group [11]. The result of the clinical pilot study was used for the sample size calculation. Samples were randomly divided into three groups using a computer-generated simple randomization method. Thirty contaminated healing abutments were used for each group.

After electrochemical cleaning, the healing abutment was rinsed with distilled water and placed in an individual plastic tube (Centrifuge tube 12-6265, SANSYO, Tokyo, Japan) containing 2 ml phloxine B peptide-staining solution (Phloxine B fluorescent dye, Sigma Aldrich, Tokyo, Japan) [12]. After staining, the healing abutment was rinsed again with distilled water and air-dried.

### Analysis methods

The following methods were used to analyze the chemical and morphological properties of contaminated surface and surface composition after electrolysis.

#### Analysis of the electrolytes before and after electrolysis

The electrolytic solutions (catholyte and anolyte) were analyzed for pH before and after electrolysis by pH meter (LAQUA D-71 pH meter, Horiba, Kyoto, Japan).

#### Evaluation of the amount of stained area (contamination) of the healing abutments

The healing abutments were photographed using a light microscope and digital capture system at × 2 magnification (SMZ800, Nikon, Tokyo, Japan). Three images were captured for each healing abutment: two images from the body of the healing abutments rotated at 180° and one image from the top. The captured photographs were digitally analyzed with ImageJ software (National Institutes of Health, Bethesda, MD, USA) to measure the stained (and contaminated) surface areas. The color threshold value was manually adjusted within the range of 0 to 250 to detect all the stained debris, while a dark background color was established in order to standardize all the measurements. This manual threshold manipulation allowed for the selection of a stained area in which the number of pixels was calculated. Surface area contamination was expressed as a fraction (%) of the total surface area by dividing the number of pixels within the selected area by the total number of pixels comprising the image.

#### Scanning electron microscopy (SEM) analysis and visual assessment

The healing abutments were evaluated using scanning electron microscopy (SEM; S-4500, Hitachi, Japan) in order to quantitatively analyze the possible surface changes caused by electrolysis. For holding the samples, standard aluminum SEM specimen mounting stubs with specially designed sample holders were used. SEM was used with a setting of 10 kV accelerating voltage and the magnification of × 1000. The samples were handled with sterilized titanium tweezers to prevent surface contamination. Left side of upper part of the body of healing abutments located below the identification letter was used as area of interest to be examined. Only images of sign of surface changes were saved, which resulted in large number of SEM pictures for each different charges and currents. Four representative images that showed the most obvious visual surface changes were selected irrespective of the number of healing abutments per electrolysis of different charges and currents. This resulted in a total of 32 (× 1000).

In order to objectively rate the surface changes, images were coded and then blindly and independently scored by three examiners (T.H., K.K., N.K.). This means that each assessor was unaware from which electrolysis treatment each image came from except for the image of the unused (control) healing abutment. The following categorical rating score proposed by Bain [13] was used: (1) smoother (less rough) than the control, (2) same as new untreated control, (3) rougher than the control, and (4) much rougher than the control.

Kendall’s coefficient of concordance was used to investigate the inter-examiner reliability in the evaluation of SEM images. There was no significant difference between the three examiners (*w* = 0.815, *P* < 0.01).

#### Energy-dispersive X-ray spectroscopy (EDS) analysis

For quantitative analysis of titanium surfaces, EDS analysis was used. The spectroscopy of the emitted X-ray photons was performed by energy-resolved X-ray analyzer (Horiba EMAX-7000, Japan) at 15 kV for 100 s with the working distance of 15 mm in the vacuum condition without conductive coating. Three different measuring areas in same size were randomly selected on the surface of each sample to examine the atomic percentage of titanium and carbon.

### Statistical analysis

The residual areas on the contaminated surfaces of the healing abutments following three different electrochemical treatments were calculated, compared, and analyzed using one-way ANOVA test. The mean roughness scores with standard deviations were calculated for each individual examiner and selected electrolytic cleaning treatment. Pairwise comparisons were used to compare the mean results of the surface roughness scores of all examiners between the different electrolytic treatments. *P* values less than 0.05 were considered statistically significant. All statistical analyses were performed using IBM SPSS Statistics for Windows, Version 21.0 (IBM, Armonk, NY, USA).

## Results

### Analysis of the electrolytes’ pH before and after electrolysis

As shown in Table 1, pH of electrolyte (catholyte) was not changed after electrolysis of different currents. However, the pH decreased after electrolysis of anodic potential of 1 A and 1.5 A in group I and II.

**Table 1:** pH after electrolysis. pH was measured after electrolysis for 5 min under different charges and current at constant 10 V. pH of original electrolyte, 7.5% NaHCO_3_, before electrolysis was 7.8

Charges and currents	Group I	Group II	Group III
−, 0.5 A	7.91	7.92	7.94
+, 0.5 A	7.02	7.03	7.02
−, 1 A	7.94	8.02	8.01
+, 1 A	6.6	6.2	7.72
−, 1.5 A	7.98	8.1	8.2
+, 1.5 A	6.7	6.8	7.92

### Evaluation of the amount of stained area (contamination) of the healing abutments

**Fig. 1:**
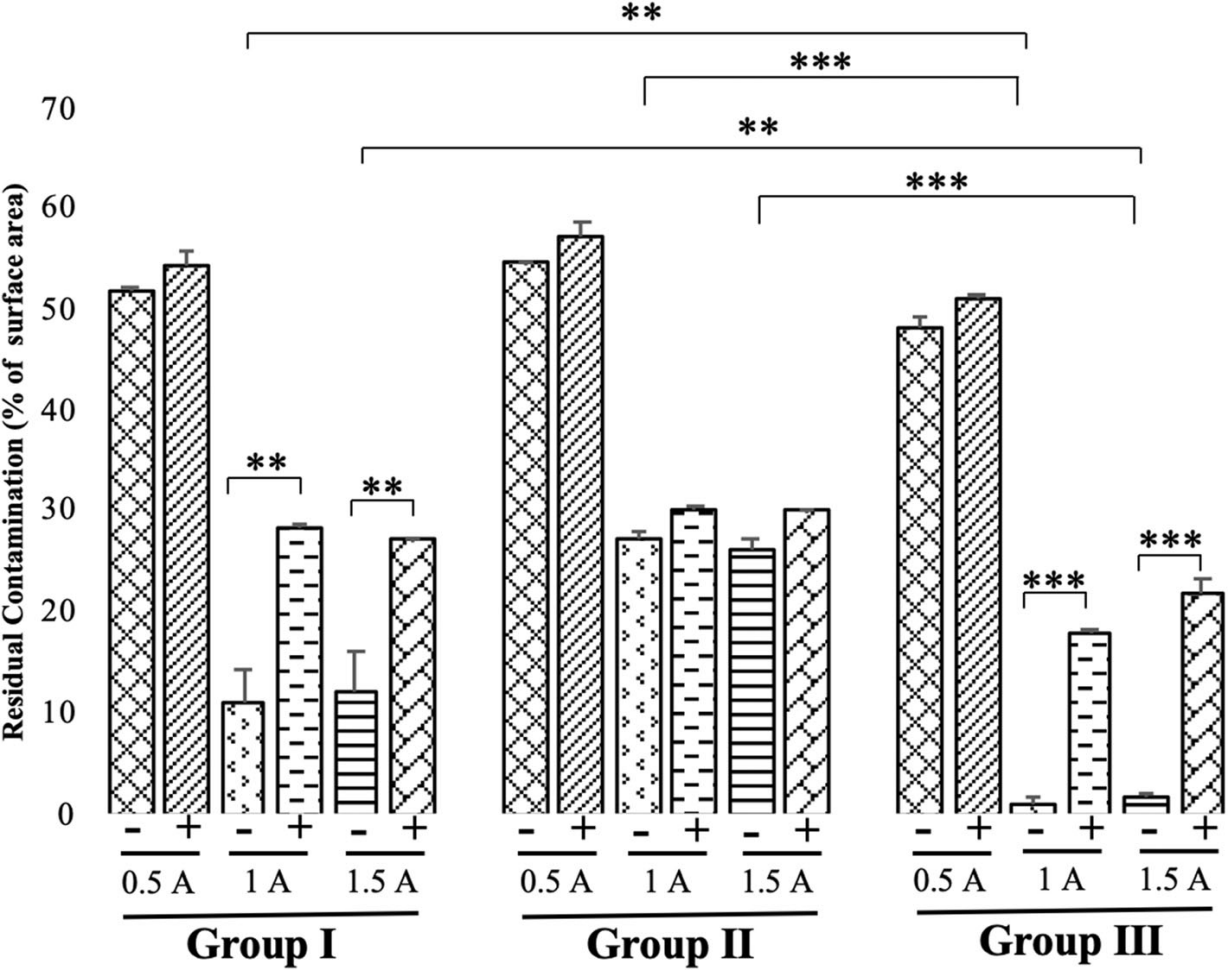
The amount of residual contamination after electrochemical treatments under different charges and currents at constant 10 V. Mean ± SD (*n* = 5). *P < 0.05, **P < 0.01, ***P < 0.005

The percentage of residual contaminated area of the healing abutments after electrochemical treatments with 0.5 A showed no significant differences between cathodic and anodic potential in group I, II, and III (Fig. 1). However, significant differences were seen between cathodic and anodic potential of 1 A and 1.5 A in both group I (*P* < 0.05) and group III (*P* < 0.01). In group I, electrochemical treatments with 1 A and 1.5 A in cathodic potential showed 11% and 12% of residual contamination, respectively. Electrochemical treatments in group II showed no significant differences in the percentage of residual contamination between 1 A and 1.5 A in cathodic and anodic potential. Although the use of 1 A and 1.5 A was more effective than 0.5 A in both cathodic and anodic potential, complete decontamination was not achieved. Among three groups, applying 1 A in cathodic potential in group III resulted in the lowest percentage of residual contamination on the healing abutments compared with group I (*P* < 0.05) and group II (*P* < 0.01). In addition, the percentage of residual contamination after applying 1.5 A in cathodic potential in group III also resulted in significantly lower than that in group I (*P* < 0.05) and group II (*P* < 0.01).

**Fig. 2:**
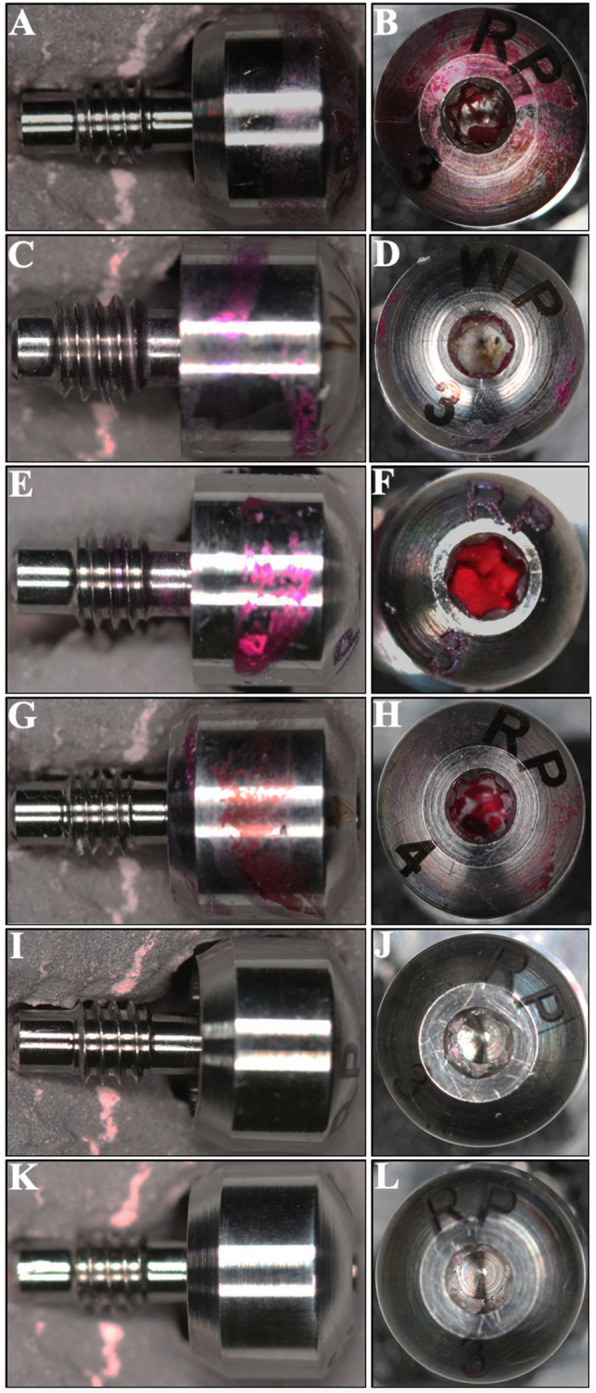
Microscopical images of the healing abutments after the electrochemical treatments under different currents at constant 10 V with different electrodes. The healing abutments were stained with phloxine B after electrolysis. Images from side (**a**, **c**, **e**, **g**, **i**, **k**) and from top (**b**, **d**, **f**, **h**, **j**, **l**). **a**, **b** 1 A group I. **c**, **d** 1.5 A group I. **e**, **f** 1 A group II. **g**, **h** 1.5 A group II. **i**, **j** 1 A group III. **k**, **l** 1.5 A group III

After applying 1 A and 1.5 A in cathodic potential in group III, the percentage of residual contamination was 1% and 2%, respectively. Microscopic views of a healing abutment stained with phloxine B after cleaning with different electrolytic cleaning treatments were shown in Fig. 2.

### Analysis of healing abutment surface roughness after electrolysis

**Fig. 3:**
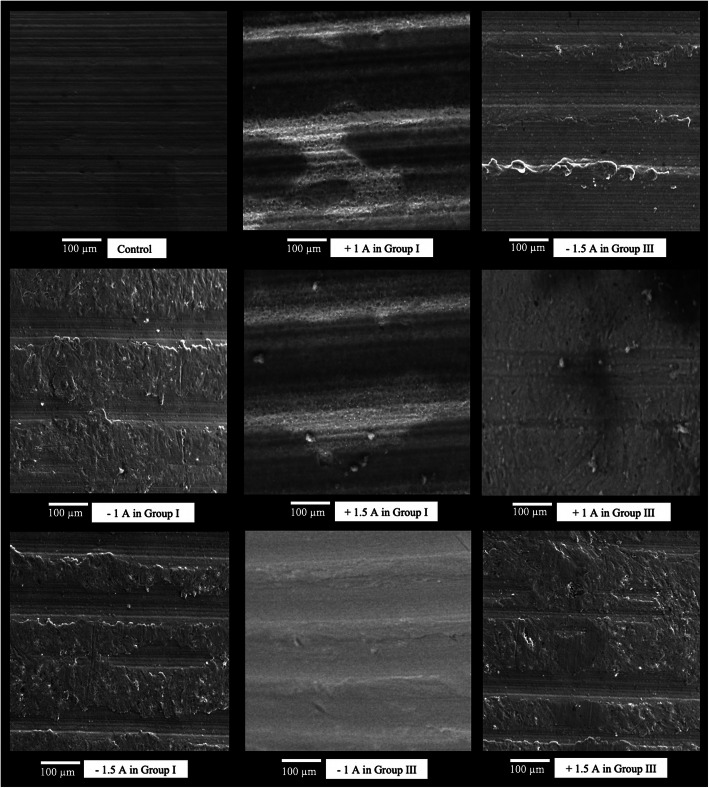
Representative SEM images of healing abutments after electrolysis of different charges and currents for 10 V and 5 min (all images × 1000 magnification)

Representative SEM images after electrolysis of two different charges (cathodic and anodic) and two different currents (1 A and 1.5 A) are presented in Fig. 3. The SEM images showed surface modification ranging from smoothening to roughening. The surfaces were between the electrolytic healing abutments and control unused healing abutment. Since the debris were covered in the surface of healing abutments especially in the region of interest, surface roughness after electrolysis in group II, low cathodic and anodic potential of group I and III were not taken into scoring for surface roughness. All 1 A and 1.5 A of cathodic and anodic potentials in both group I and III resulted in the least surface modification except 1 A of cathodic potential in group III. In group I, 1.5 A of cathodic potential induced the most alteration, followed by 1.5 A of anodic potential and the least alteration at 1 A of cathodic potential. In group III, the most surface alteration was seen after electrolysis with 1.5 A of anodic potential, followed by 1 A of anodic potential and the least change at 1 A of cathodic potential.

Then, a more objective and methodological visual assessment of the surface roughness was conducted using ranking system by Bain [13]. Table 2 summarizes the results of the scores of 4 SEM images per electrolysis of each examiner and all of the examiners combined as well as the *P* values compared to the untreated control. As shown in Table 2, the mean results showed that cathodic 1.5 A in group I scored the highest degree of surface roughness (3.92 ± 0.17) among the examined groups. In contrast, lowest degree of surface roughness scoring (2.00 ± 0.17) was seen at cathodic 1 A in group III. In addition, cathodic 1.5 A in group III scored higher surface roughness (3.67 ± 0.00) than untreated control (grade 2).

**Table 2:** Qualitative analysis of surfaces after electrolysis under different conditions. Three examiners independently scored each surface based on 4 SEM images mean ± SD

	Mean roughness score of 4 SEM images per electrolysis of different charges and currents			Mean all examiners	*P* value compared to Control
	Ex 1	Ex 2	Ex 3		
Control	2.00 ± 0.00	2.00 ± 0.00	2.00 ± 0.00	2.00 ± 0.00	-
− 1 A, group I	4.00 ± 0.00	3.00 ± 0.00	3.25 ± 0.50	3.42 ± 0.17	*
− 1.5 A, group I	4.00 ± 0.00	3.75 ± 0.50	4.00 ± 0.00	3.92 ± 0.17	*
+ 1 A, group I	3.25 ± 0.50	3.33 ± 0.58	3.75 ± 0.50	3.44 ± 0.53	*
+ 1.5 A, group I	3.25 ± 0.50	4.00 ± 0.00	3.75 ± 0.50	3.67 ± 0.33	*
− 1 A, group III	2.00 ± 0.50	2.00 ± 0.00	2.00 ± 0.00	2.00 ± 0.17	n.s.
− 1.5 A, group III	4.00 ± 0.00	3.00 ± 0.00	3.00 ± 0.00	3.33 ± 0.00	*
+ 1 A, group III	3.00 ± 0.00	4.00 ± 0.00	3.25 ± 0.50	3.42 ± 0.17	*
+ 1.5 A, group III	3.00 ± 0.00	4.00 ± 0.00	4.00 ± 0.00	3.67 ± 0.00	*

*n.s.* no significance; *P < 0.05

### Surface chemistry of clean and contaminated healing abutments

EDS analysis showed significant differences in the elemental composition of tested healing abutments. Table 3 showed the composition of tested healing abutments and unused healing abutment which are analyzed by EDS. The unused implant healing abutment contained titanium (97.56) and carbon (2.44). It was observed that all the tested healing abutments contain titanium. The highest titanium value (96.88) was seen in 1.5 A cathodic potential in group III, while the lowest titanium value (21.48) was found in 0.5 A anodic potential in group II. The debris part in the surface composition of healing abutments is represented by the presence of carbon. The highest carbon content (74.14) among all evaluated healing abutments was found in 0.5 A in anodic potential. However, 1.5 A and 1 A cathodic potential in group III showed the lowest carbon content (3.12 and 3.67) respectively. In group II, copper was observed in 0.5 A (4.38), 1 A (10.2), and 1.5 A (10.84) in anodic potential.

**Table 3:** Composition (%wt) of the surface of the healing abutment analyzed with EDS. Mean of 5 samples was presented

Groups	Charges and currents	Titanium	Carbon	Others
I	+ 0.5 A	24.77	75.23	-
	− 0.5 A	35.13	64.87	-
	+ 1 A	62.82	37.18	-
	− 1 A	84.10	15.90	-
	+ 1.5 A	63.86	36.14	-
	− 1.5 A	81.71	18.29	-
II	+ 0.5 A	21.48	74.14	Cu = 4.38
	− 0.5 A	29.72	70.28	-
	+ 1 A	50.15	39.65	Cu = 10.2
	− 1 A	69.16	30.84	-
	+ 1.5 A	51.05	38.11	Cu = 10.84
	− 1.5 A	67.43	32.57	-
III	+ 0.5 A	32.77	67.23	-
	− 0.5 A	41.04	58.96	-
	+ 1 A	75.27	24.73	-
	− 1 A	96.33	3.67	-
	+ 1.5 A	72.37	27.63	-
	− 1.5 A	96.88	3.12	-
Control	-	97.56	2.44	-

## Discussion

The current study showed that electrolysis could be an effective means to decontaminate the healing abutment surfaces with complete removal of contaminants without any surface changes at 10 V, 1 A into 5 min, cathodic potential in group III. A minimally invasive approach to remove and disinfect dental implants utilizes the fact that titanium is an electrically conducting metal and the number of adherent microorganisms on dental implants could be reduced by electrolysis [14–16]. Sodium bicarbonate NaHCO_3_ was used as an electrolyte in this study because it is readily available and safe and has no destructive effect on the titanium surface [17]. 7.5% NaHCO_3_ with SDS combination was effective to reduce the oral biofilm [18]. To our knowledge, no other reports have been used this electrolyte in different electrodes for the electrochemical cleaning performed in this study.

Early biofilm formation was found at the implant crown, which then progresses apically beyond the implant abutment junction leading to peri-implant diseases if not properly treated [19]. Routine methods used for cleaning and sterilization of used healing abutments may not results in the complete removal of contamination [12, 20]. The present study used contaminated healing abutments as “dirty” samples for electrolytic cleaning. These healing abutments would not investigate their consequences of reuse and potential to peri-implant diseases. Healing abutments are generally considered single use, although it is common practice for clinicians to clean and sterilize this component, often reusing it for economic reasons. Some companies also collect, clean, sterilize, and repackage these used components for sale [21]. However, recent studies have indicated that some of these components may not be as clean or sterilized as previously thought, and did question their safety for reuse [22, 23].

Phloxine B can be used to detect any remaining contamination on the cleaned surface of healing abutments. Phloxine B is a fluorescein derivative stain used to identify proteins and peptides [24]. Therefore, in this study, phloxine B staining was used to detect residual contamination on the surface of the healing abutments.

Previous studies [10, 22] also pointed out that low direct current can kill oral bacteria forming in biofilm. Although charging the implant surface with current can kill the bacteria, organic residues still remain adhering on the surface. Infected implants present carbon-based contaminants and considerable changes in titanium surfaces composition even after sterilization [25]. This could be the reason why new bone formation on previously contaminated implant surfaces, also known as re-osseointegration, remains unknown [26, 27]. Therefore, complete decontamination around infected implant surfaces requires the clinical attention to achieve implant success. Electrolytic cleaning needs mechanical cleaning for complete removal of organic contaminants to achieve re-osseointegration in infected implants [28]. Without any mechanical cleaning, the optimized electrochemical treatment was able to achieve complete decontamination on the contaminated metal surface in the present study. Biofilm-like structure was not seen in all tested healing abutments. This could be due to the effectiveness of using SDS as a presoaking detergent and the use of chemical detergents may increase the efficacy of the cleaning procedure by dissolving debris and decreasing surface tension [29].

Anodic potentials were found to inactive bacteria and eliminate their biomolecules by generating bactericidal oxidative species through the following electrolytic reactions [30].

1. 2H_2_O = O_2_ + 4H^+^ + 4e^−^
2. H_2_O = HO + H^+^ + e^−^

Based on previous study [31], anodic potentials increased Ti_2_O surface concentration that probably suggest oxidation of titanium, and high level of contamination was seen. Similarly, in our study, this potential could have bactericidal effect but the adsorption and oxidation of negatively charged bacteria on anodic surface result in accumulation of dead bacteria that blocks further reactions and limits the removal of contaminants. As EDS analysis was used in this study, Ti_2_O level can't identify. However, copper deposition was formed on the sample after electrolysis of anodic potential in group II. It is believed that anodic current was preferentially supplied by the electrolysis of water, which occurred on the surface of the sample because deposition attained electroconductivity. Thus, it was confirmed that copper could be incorporated into the titanium surface sample, to some extent, with cathodic current applied in this study.

The application of voltage to reduce bacterial load has been described several times in the literature. Mohn et al. [7] evaluated the disinfection of biofilm-contaminated implant surfaces using low direct currents. The application of a direct anodic current of at least 7.5 mA for 15 min was able to eliminate an *Escherichia coli* biofilm on implant surfaces. However, the voltage (ranged from 4 to 20 V) was used in their study. In another study [9], the removal of multispecies biofilm on implant disks was achieved with the application of 10 mA anodic direct current for 10 min. Though, this current seemed to alter the titanium surfaces (blue discoloration) causing delay in maturation of osteoblasts growing on them [32], also generated a high voltage (ranged between 11 and 19 V). In addition, electrolytic cleaning with a voltage of 6 V into 5 min proved to achieve the adequate disinfection of biofilm-contaminated dental implants [33]. Therefore, a fixed voltage of 10 V applied for 5 min was used in the present study.

Although stainless steel electrode retained its physicochemical properties after electrolytic biofilm removal, it can undergo corrosion at the anodic potential [34]. Copper electrode has been used in many electrochemical applications because of its corrosion resistance properties and electroconductivity [35]. Carbon electrodes are used in electrolysis due to their competence as a conductor and the number of free electrons they have available for transfer [36]. As the cleaning effect using different electrodes were observed in the current study without observing the corrosion potential on the different electrodes, the best cleaning effect was achieved using two carbon electrodes at cathodic potential.

According to the previous studies [7, 9], complete killing of bacteria was seen at anode with low current. However, in this study, complete removal of contaminant was seen in electrolysis after cathodic potential 1 A and 1.5 A in group III. This complete removing action can be attributed to the alkaline environment generated at cathodic potential. Moreover, decrease in electrolytes’ pH after electrolysis with anodic potential 1 A and 1.5 A in group II was seen. This could be attributed to the surface destructive process and influence the biocompatibility of commercially pure titanium and titanium alloy surface [37].

The use of an electric current more than 1100 mA has a potential to alter the titanium surface [16, 38]. Cathodic potentials can generate electro-repulsive forces between the negatively charged surface and bacteria, resulting in their detachment [39]. It can also induce water electrolysis that produce hydrogen gas and increased pH (alkaline) as stated above. The alkaline pH has bactericidal effects mainly through hydrolysis of the bacterial polysaccharide matrix, whereas the generated H_2_ gas bubbles adjuncts the detachment of surface contaminants and bacteria [40]. Therefore, cathodic potential 1 A in group III did not result in a significantly greater surface roughness compared to the control suggesting that there was no visible surface alternation on the surfaces. Although using cathodic potential 1.5 A in group III can also be removed the contamination completely, significantly higher surface roughness compared to other groups was seen. However, the subjective nature of the scoring method could potentially influence these results. The findings in this study showed that in spite of some of the differences in surface changes of dental implant healing abutments caused by electrolysis, there were no significant differences between that cathodic potential 1 A and 1.5 A in group III in the percentage of residual contamination.

Although EDS analysis was performed in three areas on each sample, the atomic percentage of carbon on the contaminated area was higher than that on the clean area and the atomic percentage of titanium was lower on the contaminated areas than on the clean area except cathodic potential 1 A and 1.5 A in group III. The possible sources of carbon contamination in the EDS analysis are not yet confirmed [41]. In our study, carbon peak was not detected in the surfaces treated with cathodic potential 1 A and 1.5 A in group III suggesting that there was no organic contamination on the surfaces. Copper was seen on the sample after electrolysis of anodic potential in group II suggesting that using the copper electrode at anodic potential has fairly limited potential for electrolytic cleaning performed in this study because using copper as electrode at anodic potential may initiate the electroplating process in which copper particles can deposit on the metal surface.

The limitations of the current study included only titanium healing abutments were evaluated after electrolysis; other contaminated components are considered in need of future evaluation. Although electrochemical treatment with 7.5% NaHCO_3_ in group III had no detrimental effects to the surface, further investigation should aim to access the current approach in an animal model for peri-implantitis. Our findings showed that using 7.5% NaHCO_3_ electrolyte in a short time (5 min) of cathodic potential of 1 A in group III can provide an alternative treatment system for cleaning the contaminated healing abutments; SEM showed no visible alterations on the surface of the healing abutments and the EDS analysis confirmed no signs of organic contamination on the surfaces.

## Conclusion

Despite the limitation of this study, the present results suggested that electrolysis of using carbon as electrodes, placing the contaminated healing abutments on cathode and applying an electric current of 1 A at constant 10 V in 7.5% sodium bicarbonate could completely remove organic contaminants from the surfaces. The findings of the present study could prompt further research into this newly established method. In particular, this optimized electrochemical cleaning method seems to be well worth investigation for the clinical management of peri-implant infections.

## Data Availability

All data and materials are available from the corresponding author.

## Abbreviations

SDS: Sodium dodecyl sulfate (Fujifilm Wako Pure Chemical Corp., Tokyo, Japan)
NaHCO_3_: Sodium bicarbonate (LEC Chemical Corp., Tokyo, Japan)
SEM: Scanning electron microscopy (S-4500, Hitachi, Japan)
EDS: Energy-dispersive X-ray spectroscopy (Horiba EMAX-7000, Japan)
Ti_2_O: Titanium dioxide
H_2_O: Water
H: Hydrogen
O_2_: Oxygen
OH^-^: Hydroxide
e: Electron
